# 5-Benzoyl-*N*,4-diphenyl-4,5-dihydro-1*H*-pyrazole-3-carboxamide

**DOI:** 10.1107/S1600536809034035

**Published:** 2009-08-29

**Authors:** Long He

**Affiliations:** a College of Chemistry and Chemical Engineering, China West Normal University, Nanchong 637002, People’s Republic of China

## Abstract

The title compound, C_23_H_19_N_3_O_2_, was synthesized by the 1,3-dipolar cyclo­addition reaction of *N*-phenyl-α-diazo­acetamide and chalcone. In the mol­ecule, the pyrazoline ring assumes an envelope conformation. Weak inter­molecular C—H⋯O hydrogen bonding is present in the crystal structure.

## Related literature

For the 1,3-dipolar cyclo­addition reaction, see: Grigg (1995 ▶). For applications of pyrazoline and its derivatives, see: Dhal *et al.* (1975 ▶); Lombardino & Ottemes (1981 ▶); Parmar *et al.* (1974 ▶); Rawal *et al.* (1963 ▶).
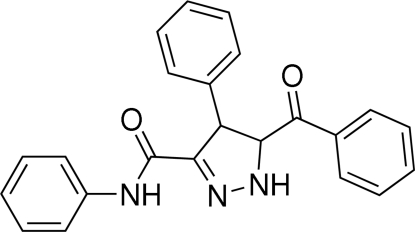


## Experimental

### 

#### Crystal data

C_23_H_19_N_3_O_2_
*M* *_r_* = 369.41
Monoclinic, 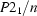

*a* = 5.809 (3) Å
*b* = 10.717 (5) Å
*c* = 29.428 (7) Å
β = 92.753 (5)°
*V* = 1829.9 (13) Å^3^
*Z* = 4
Cu *K*α radiation
μ = 0.70 mm^−1^
*T* = 293 K
0.36 × 0.24 × 0.20 mm

#### Data collection

Oxford Diffraction Gemini S Ultra diffractometer
Absorption correction: multi-scan (*CrysAlis RED*; Oxford Diffraction, 2007 ▶) *T*_min_ = 0.786, *T*_max_ = 0.873
27720 measured reflections
2929 independent reflections
2390 reflections with *I* > 2σ(*I*)
*R*_int_ = 0.031

#### Refinement

*R*[*F* ^2^ > 2σ(*F* ^2^)] = 0.034
*wR*(*F* ^2^) = 0.066
*S* = 1.00
2929 reflections
261 parameters
2 restraints
H atoms treated by a mixture of independent and constrained refinement
Δρ_max_ = 0.16 e Å^−3^
Δρ_min_ = −0.15 e Å^−3^

### 

Data collection: *CrysAlis CCD* (Oxford Diffraction, 2007 ▶); cell refinement: *CrysAlis RED* (Oxford Diffraction, 2007 ▶); data reduction: *CrysAlis RED*; program(s) used to solve structure: *SHELXS97* (Sheldrick, 2008 ▶); program(s) used to refine structure: *SHELXL97* (Sheldrick, 2008 ▶); molecular graphics: *ORTEP-3* (Farrugia, 1997 ▶); software used to prepare material for publication: *SHELXL97*.

## Supplementary Material

Crystal structure: contains datablocks global, I. DOI: 10.1107/S1600536809034035/xu2571sup1.cif

Structure factors: contains datablocks I. DOI: 10.1107/S1600536809034035/xu2571Isup2.hkl

Additional supplementary materials:  crystallographic information; 3D view; checkCIF report

## Figures and Tables

**Table 1:** Hydrogen-bond geometry (Å, °)

*D*—H⋯*A*	*D*—H	H⋯*A*	*D*⋯*A*	*D*—H⋯*A*
C3—H3⋯O1^i^	0.93	2.56	3.468 (4)	166
C8—H8⋯O1^i^	0.98	2.50	3.475 (4)	173

Symmetry code: (i) 
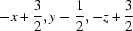
.

**Fig. 1.**
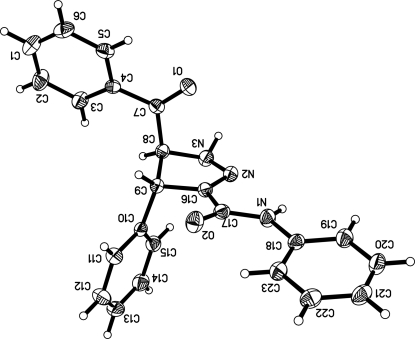
The molecular structure of (I) with 30% probability displacement ellipsoids (arbitrary spheres for H atoms).

## supplementary crystallographic information

### Comment

The 1,3-dipolar cycloaddition reaction is one of the most efficient and widely used methods for the synthesis of nitrogen-containing five-membered heterocycles (Grigg, 1995). As important and useful five-membered heterocyclic compounds, pyrazoline and its derivatives were found to possess antifungal (Dhal *et al.*, 1975), immunosuppressive (Lombardino *et al.*,1981), psychoanaleptic (Parmar *et al.* 1974), and antiviral (Rawal *et al.* 1963) activities. We report herein the crystal structure of the title compound.

The molecular structure of (I) is shown in Fig. 1. In the molecule the dihydropyrazole ring assumes an envelope conformation. The C6-benene ring and C15-benene ring make dihedral angles of 82.08 (7)° and 84.78 (7)° with respect to the C23-benene ring. The dihedral angle between the C6-benzene and C15-benene ring is 71.39 (7)°. Intermolecular weak C—H···O hydrogen bonding is present in the crystal structure (Table 1).

### Experimental

*N*-Phenyl-alfa-diazoacetamide (0.035 g, 0.2 mmol) and chalcone (0.042 g, 0.2 mmol) and 1,4-diaza-bicyclo[2.2.2]octan (0.02 g, 0.2 mmol) were dissolved in toluene (2 mL). The solution was warmed to 323 K, and the solution was stirred for 2 h. After removal of solvent under reduced pressure, the residue was purified through column chromatography on silica gel to give target compound. Colourless single crystals suitable for X-ray diffraction were obtained by recrystallization from ethanol.

### Refinement

Imino H atoms were located in a difference Fourier map and were refined isotropically. Other H atoms were placed in calculated positions with C—H = 0.93  or 0.98 Å, and refined using a riding model, with *U*_iso_(H) =1.2*U*_eq_(C).

### Crystal data

**Table:**

C_23_H_19_N_3_O_2_	*F*(000) = 776
*M**_r_* = 369.41	*D*_x_ = 1.341 Mg m^−^^3^
Monoclinic, *P*2_1_/*n*	Cu *K*α radiation, λ = 1.54184 Å
Hall symbol: -P 2yn	Cell parameters from 11458 reflections
*a* = 5.809 (3) Å	θ = 3.0–62.7°
*b* = 10.717 (5) Å	µ = 0.70 mm^−^^1^
*c* = 29.428 (7) Å	*T* = 293 K
β = 92.753 (5)°	Block, colorless
*V* = 1829.9 (13) Å^3^	0.36 × 0.24 × 0.20 mm
*Z* = 4	

### Data collection

**Table:**

Oxford Diffraction Gemini S Ultra diffractometer	2929 independent reflections
Radiation source: Ultra (Cu) X-ray Source	2390 reflections with *I* > 2σ(*I*)
mirror	*R*_int_ = 0.031
Detector resolution: 15.9149 pixels mm^-1^	θ_max_ = 62.7°, θ_min_ = 3.0°
ω scans	*h* = −6→6
Absorption correction: multi-scan (*CrysAlis RED*; Oxford Diffraction, 2007)	*k* = −12→12
*T*_min_ = 0.786, *T*_max_ = 0.873	*l* = −33→33
27720 measured reflections	

### Refinement

**Table:**

Refinement on *F*^2^	Primary atom site location: structure-invariant direct methods
Least-squares matrix: full	Secondary atom site location: difference Fourier map
*R*[*F*^2^ > 2σ(*F*^2^)] = 0.034	Hydrogen site location: inferred from neighbouring sites
*wR*(*F*^2^) = 0.066	H atoms treated by a mixture of independent and constrained refinement
*S* = 1.00	*w* = 1/[σ^2^(*F*_o_^2^) + (0.0012*P*)^2^ + 1.114*P*] where *P* = (*F*_o_^2^ + 2*F*_c_^2^)/3
2929 reflections	(Δ/σ)_max_ = 0.001
261 parameters	Δρ_max_ = 0.16 e Å^−^^3^
2 restraints	Δρ_min_ = −0.15 e Å^−^^3^

### Special details

**Table:**

Geometry. All esds (except the esd in the dihedral angle between two l.s. planes) are estimated using the full covariance matrix. The cell esds are taken into account individually in the estimation of esds in distances, angles and torsion angles; correlations between esds in cell parameters are only used when they are defined by crystal symmetry. An approximate (isotropic) treatment of cell esds is used for estimating esds involving l.s. planes.
Refinement. Refinement of *F*^2^ against ALL reflections. The weighted *R*-factor *wR* and goodness of fit *S* are based on *F*^2^, conventional *R*-factors *R* are based on *F*, with *F* set to zero for negative *F*^2^. The threshold expression of *F*^2^ > σ(*F*^2^) is used only for calculating *R*-factors(gt) *etc*. and is not relevant to the choice of reflections for refinement. *R*-factors based on *F*^2^ are statistically about twice as large as those based on *F*, and *R*- factors based on ALL data will be even larger.

### Fractional atomic coordinates and isotropic or equivalent isotropic displacement parameters (Å^2^)

**Table:**

	*x*	*y*	*z*	*U*_iso_*/*U*_eq_	
O2	0.5592 (2)	0.44501 (12)	0.90247 (4)	0.0526 (3)	
N1	0.9367 (3)	0.50816 (14)	0.90580 (5)	0.0445 (4)	
N2	1.0040 (2)	0.35676 (13)	0.83258 (4)	0.0414 (3)	
O1	0.7319 (2)	0.41770 (11)	0.74559 (4)	0.0547 (3)	
C4	0.4434 (3)	0.27270 (15)	0.72408 (5)	0.0351 (4)	
C18	0.9446 (3)	0.59203 (15)	0.94313 (5)	0.0397 (4)	
N3	1.0026 (2)	0.26477 (14)	0.79899 (5)	0.0425 (4)	
C9	0.6458 (3)	0.25420 (14)	0.83326 (5)	0.0325 (3)	
H9	0.4906	0.2885	0.8277	0.039*	
C10	0.6433 (3)	0.14494 (14)	0.86613 (5)	0.0336 (4)	
C8	0.7622 (3)	0.22873 (15)	0.78770 (5)	0.0350 (4)	
H8	0.7501	0.1407	0.7789	0.042*	
C17	0.7560 (3)	0.43921 (15)	0.88959 (5)	0.0388 (4)	
C3	0.3815 (3)	0.14837 (16)	0.71876 (6)	0.0474 (4)	
H3	0.4705	0.0863	0.7330	0.057*	
C16	0.8112 (3)	0.35355 (14)	0.85194 (5)	0.0357 (4)	
C7	0.6522 (3)	0.31416 (15)	0.75101 (5)	0.0370 (4)	
C15	0.8272 (3)	0.06224 (16)	0.87032 (6)	0.0436 (4)	
H15	0.9505	0.0706	0.8515	0.052*	
C23	0.7730 (3)	0.59886 (16)	0.97404 (6)	0.0461 (4)	
H23	0.6404	0.5508	0.9699	0.055*	
C14	0.8282 (3)	−0.03262 (17)	0.90231 (7)	0.0540 (5)	
H14	0.9514	−0.0880	0.9047	0.065*	
C20	1.1647 (4)	0.74369 (18)	0.98647 (6)	0.0554 (5)	
H20	1.2964	0.7925	0.9906	0.067*	
C21	0.9955 (4)	0.75011 (18)	1.01741 (6)	0.0540 (5)	
H21	1.0125	0.8029	1.0424	0.065*	
C6	0.1133 (3)	0.3315 (2)	0.67649 (6)	0.0572 (5)	
H6	0.0228	0.3932	0.6624	0.069*	
C11	0.4622 (3)	0.13108 (16)	0.89459 (6)	0.0447 (4)	
H11	0.3377	0.1856	0.8921	0.054*	
C5	0.3068 (3)	0.36379 (17)	0.70260 (6)	0.0471 (4)	
H5	0.3467	0.4474	0.7059	0.056*	
C19	1.1410 (3)	0.66527 (17)	0.94917 (6)	0.0493 (5)	
H19	1.2560	0.6616	0.9283	0.059*	
C22	0.8001 (3)	0.67782 (18)	1.01115 (6)	0.0524 (5)	
H22	0.6854	0.6821	1.0321	0.063*	
C2	0.1871 (3)	0.11665 (19)	0.69215 (7)	0.0597 (5)	
H2	0.1468	0.0332	0.6884	0.072*	
C13	0.6475 (4)	−0.04517 (19)	0.93055 (7)	0.0585 (5)	
H13	0.6489	−0.1086	0.9521	0.070*	
C1	0.0535 (3)	0.2077 (2)	0.67127 (7)	0.0588 (5)	
H1	−0.0773	0.1858	0.6536	0.071*	
C12	0.4647 (4)	0.03661 (19)	0.92674 (6)	0.0569 (5)	
H12	0.3426	0.0284	0.9458	0.068*	
H4	1.072 (2)	0.4961 (18)	0.8932 (6)	0.055 (6)*	
H18	1.081 (3)	0.2914 (16)	0.7754 (5)	0.052 (6)*	

### Atomic displacement parameters (Å^2^)

**Table:**

	*U*^11^	*U*^22^	*U*^33^	*U*^12^	*U*^13^	*U*^23^
O2	0.0470 (8)	0.0564 (8)	0.0546 (8)	−0.0019 (6)	0.0049 (6)	−0.0169 (6)
N1	0.0482 (10)	0.0448 (9)	0.0408 (8)	−0.0093 (7)	0.0058 (7)	−0.0109 (7)
N2	0.0417 (9)	0.0438 (8)	0.0385 (8)	−0.0059 (7)	−0.0007 (7)	−0.0024 (6)
O1	0.0634 (9)	0.0401 (7)	0.0600 (8)	−0.0133 (6)	−0.0054 (6)	0.0091 (6)
C4	0.0389 (9)	0.0372 (9)	0.0296 (8)	0.0019 (7)	0.0045 (7)	0.0002 (7)
C18	0.0509 (11)	0.0331 (9)	0.0345 (9)	−0.0008 (8)	−0.0038 (8)	−0.0024 (7)
N3	0.0363 (8)	0.0521 (9)	0.0394 (8)	−0.0017 (7)	0.0055 (7)	−0.0060 (7)
C9	0.0314 (9)	0.0322 (8)	0.0340 (8)	0.0003 (7)	0.0005 (7)	−0.0013 (7)
C10	0.0373 (9)	0.0311 (8)	0.0320 (8)	−0.0046 (7)	−0.0033 (7)	−0.0025 (7)
C8	0.0362 (9)	0.0339 (9)	0.0347 (8)	−0.0004 (7)	0.0007 (7)	−0.0035 (7)
C17	0.0495 (11)	0.0323 (9)	0.0344 (9)	−0.0019 (8)	−0.0009 (8)	0.0007 (7)
C3	0.0482 (11)	0.0372 (10)	0.0557 (11)	0.0059 (8)	−0.0086 (9)	−0.0029 (8)
C16	0.0402 (10)	0.0318 (9)	0.0348 (9)	−0.0027 (7)	0.0000 (7)	0.0003 (7)
C7	0.0424 (10)	0.0350 (9)	0.0340 (9)	0.0003 (8)	0.0068 (7)	−0.0022 (7)
C15	0.0411 (10)	0.0415 (10)	0.0479 (10)	−0.0001 (8)	−0.0021 (8)	0.0024 (8)
C23	0.0501 (11)	0.0460 (10)	0.0420 (10)	−0.0053 (9)	0.0005 (8)	−0.0055 (8)
C14	0.0543 (12)	0.0438 (11)	0.0621 (12)	0.0029 (9)	−0.0148 (10)	0.0086 (9)
C20	0.0600 (13)	0.0489 (11)	0.0562 (12)	−0.0122 (10)	−0.0095 (10)	−0.0089 (9)
C21	0.0704 (14)	0.0464 (11)	0.0441 (10)	0.0033 (10)	−0.0085 (10)	−0.0125 (9)
C6	0.0568 (13)	0.0617 (13)	0.0520 (12)	0.0123 (10)	−0.0089 (10)	0.0111 (10)
C11	0.0454 (11)	0.0433 (10)	0.0456 (10)	−0.0008 (8)	0.0049 (8)	0.0025 (8)
C5	0.0542 (12)	0.0412 (10)	0.0457 (10)	0.0020 (9)	0.0014 (9)	0.0092 (8)
C19	0.0525 (12)	0.0487 (11)	0.0467 (10)	−0.0082 (9)	0.0028 (9)	−0.0064 (9)
C22	0.0597 (13)	0.0557 (12)	0.0419 (10)	0.0039 (10)	0.0030 (9)	−0.0082 (9)
C2	0.0556 (13)	0.0461 (11)	0.0758 (14)	0.0002 (10)	−0.0145 (11)	−0.0150 (10)
C13	0.0739 (15)	0.0499 (12)	0.0503 (11)	−0.0119 (11)	−0.0105 (10)	0.0170 (9)
C1	0.0491 (12)	0.0708 (14)	0.0549 (12)	0.0069 (10)	−0.0138 (9)	−0.0100 (10)
C12	0.0642 (14)	0.0582 (12)	0.0490 (11)	−0.0101 (11)	0.0111 (10)	0.0104 (10)

### Geometric parameters (Å, °)

**Table:**

O2—C17	1.223 (2)	C15—C14	1.385 (2)
N1—C17	1.352 (2)	C15—H15	0.9300
N1—C18	1.418 (2)	C23—C22	1.385 (2)
N1—H4	0.895 (9)	C23—H23	0.9300
N2—C16	1.281 (2)	C14—C13	1.376 (3)
N2—N3	1.3959 (19)	C14—H14	0.9300
O1—C7	1.216 (2)	C20—C21	1.373 (3)
C4—C3	1.387 (2)	C20—C19	1.384 (2)
C4—C5	1.391 (2)	C20—H20	0.9300
C4—C7	1.485 (2)	C21—C22	1.379 (3)
C18—C23	1.383 (2)	C21—H21	0.9300
C18—C19	1.390 (2)	C6—C5	1.375 (3)
N3—C8	1.471 (2)	C6—C1	1.379 (3)
N3—H18	0.896 (9)	C6—H6	0.9300
C9—C10	1.519 (2)	C11—C12	1.385 (2)
C9—C16	1.519 (2)	C11—H11	0.9300
C9—C8	1.555 (2)	C5—H5	0.9300
C9—H9	0.9800	C19—H19	0.9300
C10—C11	1.384 (2)	C22—H22	0.9300
C10—C15	1.389 (2)	C2—C1	1.373 (3)
C8—C7	1.532 (2)	C2—H2	0.9300
C8—H8	0.9800	C13—C12	1.377 (3)
C17—C16	1.486 (2)	C13—H13	0.9300
C3—C2	1.385 (3)	C1—H1	0.9300
C3—H3	0.9300	C12—H12	0.9300
			
C17—N1—C18	127.97 (16)	C14—C15—H15	119.8
C17—N1—H4	117.2 (12)	C10—C15—H15	119.8
C18—N1—H4	114.6 (13)	C18—C23—C22	119.52 (18)
C16—N2—N3	108.71 (13)	C18—C23—H23	120.2
C3—C4—C5	118.93 (16)	C22—C23—H23	120.2
C3—C4—C7	123.26 (15)	C13—C14—C15	120.24 (18)
C5—C4—C7	117.80 (15)	C13—C14—H14	119.9
C23—C18—C19	119.96 (16)	C15—C14—H14	119.9
C23—C18—N1	123.06 (16)	C21—C20—C19	120.62 (19)
C19—C18—N1	116.91 (16)	C21—C20—H20	119.7
N2—N3—C8	108.57 (13)	C19—C20—H20	119.7
N2—N3—H18	109.8 (12)	C20—C21—C22	119.59 (18)
C8—N3—H18	114.9 (12)	C20—C21—H21	120.2
C10—C9—C16	109.58 (13)	C22—C21—H21	120.2
C10—C9—C8	115.57 (13)	C5—C6—C1	119.95 (18)
C16—C9—C8	98.12 (12)	C5—C6—H6	120.0
C10—C9—H9	111.0	C1—C6—H6	120.0
C16—C9—H9	111.0	C10—C11—C12	120.56 (18)
C8—C9—H9	111.0	C10—C11—H11	119.7
C11—C10—C15	118.77 (16)	C12—C11—H11	119.7
C11—C10—C9	119.93 (15)	C6—C5—C4	120.72 (18)
C15—C10—C9	121.17 (15)	C6—C5—H5	119.6
N3—C8—C7	111.19 (13)	C4—C5—H5	119.6
N3—C8—C9	101.93 (12)	C20—C19—C18	119.61 (18)
C7—C8—C9	108.60 (13)	C20—C19—H19	120.2
N3—C8—H8	111.6	C18—C19—H19	120.2
C7—C8—H8	111.6	C21—C22—C23	120.70 (19)
C9—C8—H8	111.6	C21—C22—H22	119.6
O2—C17—N1	125.75 (16)	C23—C22—H22	119.6
O2—C17—C16	120.06 (15)	C1—C2—C3	120.43 (19)
N1—C17—C16	114.16 (16)	C1—C2—H2	119.8
C2—C3—C4	119.96 (17)	C3—C2—H2	119.8
C2—C3—H3	120.0	C14—C13—C12	119.73 (18)
C4—C3—H3	120.0	C14—C13—H13	120.1
N2—C16—C17	122.66 (15)	C12—C13—H13	120.1
N2—C16—C9	114.14 (14)	C2—C1—C6	120.00 (19)
C17—C16—C9	123.20 (15)	C2—C1—H1	120.0
O1—C7—C4	120.64 (15)	C6—C1—H1	120.0
O1—C7—C8	119.30 (15)	C13—C12—C11	120.24 (19)
C4—C7—C8	119.99 (14)	C13—C12—H12	119.9
C14—C15—C10	120.45 (18)	C11—C12—H12	119.9
			
C17—N1—C18—C23	11.9 (3)	C3—C4—C7—C8	−22.0 (2)
C17—N1—C18—C19	−171.13 (17)	C5—C4—C7—C8	159.26 (15)
C16—N2—N3—C8	18.98 (18)	N3—C8—C7—O1	−22.7 (2)
C16—C9—C10—C11	102.37 (17)	C9—C8—C7—O1	88.68 (18)
C8—C9—C10—C11	−147.97 (15)	N3—C8—C7—C4	160.37 (13)
C16—C9—C10—C15	−73.52 (19)	C9—C8—C7—C4	−88.26 (17)
C8—C9—C10—C15	36.1 (2)	C11—C10—C15—C14	0.3 (2)
N2—N3—C8—C7	86.75 (16)	C9—C10—C15—C14	176.24 (15)
N2—N3—C8—C9	−28.81 (16)	C19—C18—C23—C22	−0.8 (3)
C10—C9—C8—N3	−90.48 (15)	N1—C18—C23—C22	176.05 (17)
C16—C9—C8—N3	25.84 (14)	C10—C15—C14—C13	−0.6 (3)
C10—C9—C8—C7	152.07 (13)	C19—C20—C21—C22	−0.1 (3)
C16—C9—C8—C7	−91.60 (14)	C15—C10—C11—C12	0.2 (3)
C18—N1—C17—O2	6.2 (3)	C9—C10—C11—C12	−175.83 (16)
C18—N1—C17—C16	−175.71 (16)	C1—C6—C5—C4	−0.2 (3)
C5—C4—C3—C2	0.4 (3)	C3—C4—C5—C6	0.0 (3)
C7—C4—C3—C2	−178.35 (17)	C7—C4—C5—C6	178.84 (16)
N3—N2—C16—C17	179.69 (14)	C21—C20—C19—C18	−0.3 (3)
N3—N2—C16—C9	0.10 (19)	C23—C18—C19—C20	0.7 (3)
O2—C17—C16—N2	169.36 (16)	N1—C18—C19—C20	−176.31 (17)
N1—C17—C16—N2	−8.8 (2)	C20—C21—C22—C23	0.0 (3)
O2—C17—C16—C9	−11.1 (2)	C18—C23—C22—C21	0.4 (3)
N1—C17—C16—C9	170.74 (14)	C4—C3—C2—C1	−0.6 (3)
C10—C9—C16—N2	103.60 (16)	C15—C14—C13—C12	0.4 (3)
C8—C9—C16—N2	−17.29 (17)	C3—C2—C1—C6	0.4 (3)
C10—C9—C16—C17	−75.98 (19)	C5—C6—C1—C2	0.0 (3)
C8—C9—C16—C17	163.12 (14)	C14—C13—C12—C11	0.1 (3)
C3—C4—C7—O1	161.11 (17)	C10—C11—C12—C13	−0.4 (3)
C5—C4—C7—O1	−17.6 (2)		

### Hydrogen-bond geometry (Å, °)

**Table:**

*D*—H···*A*	*D*—H	H···*A*	*D*···*A*	*D*—H···*A*
C3—H3···O1^i^	0.93	2.56	3.468 (4)	166
C8—H8···O1^i^	0.98	2.50	3.475 (4)	173

Symmetry codes: (i) −*x*+3/2, *y*−1/2, −*z*+3/2.
